# Cytotoxic and Antioxidant Activity of a Set of Hetero Bicylic Methylthiadiazole Hydrazones: A Structure-Activity Study

**Published:** 2015

**Authors:** Paulrasu Kodisundaram, Arul Duraikannu, Thirunavukkarasu Balasankar, Pravin Sundarao Ambure, Kunal Roy

**Affiliations:** 1 *Department of Chemistry, Annamalai University, Annamalai Nagar-608002, Tamilnadu, India. *; 2 *Department of Clinical Neurosciences, University of Calgary, Calgary, Alberta T2N 4N1, Canada.*; 3 *Chemistry section, FEAT, Annamalai University, Annamalai nagar-608002, Tamilnadu, India.*; 4 *Drug Theoretics and Cheminformatics Laboratory, Department of Pharmaceutical Technology, Jadavpur University, Jadavpur, Kolkata, 700032, India.*; 5 *Manchester Institute of Biotechnology, University of Manchester, Manchester M1 7DN, Great Britain.*

**Keywords:** Antioxidant, anticancer, 3- azabicylonones, hydrazones, cytotoxicity

## Abstract

The current study highlights the *in vitro* antioxidant and antitumor activity of the previously-synthesized hydrazone derivatives against various free radicals and human cancer cell lines, respectively. The anticancer efficacies of the compound were tested by measuring cytotoxicity in cancer cell lines HeLa, A549, and non-cancerous NL20 cells. Compounds possessing electron-donor methoxy and methyl substitutions at the para position of the phenyl ring moiety showed a concentration dependent free radical scavenging effects. The free radical-scavenging potential of synthetic compounds 11 and 14 may have significant impact on the prevention of free radical-induced oxidative stress and carcinogenesis. The results from cytotoxicity and cell migration assay showed that the substitution of electron-withdrawing fluoro, chloro and bromo functional groups induced a significant (P< 0.001) loss of cell viability and inhibited the invasive potential of the human cancer cells. Additionally, these compounds showed significantly (P< 0.05) a less toxicity toward non-cancerous NL20 cells. Docking studies revealed interactions of compound 10 with p38*α *MAP kinase, which may be responsible of its anti-invasive and anti-proliferative effects.

Reactive oxygen species (ROS) are essential for an organism’s vital activities such as the regulation of cell proliferation, intracellular signaling and synthesis of biologically active compounds, and energy. Excessive production of ROS causes oxidative stress and chronic diseases such as cardiovascular disease, diabetes and cancer. ROS are known to directly interact with all types of biomolecules, including proteins, lipids and DNA (1-3). ROS can easily react with membrane lipids, causing an alteration of membrane permeability. With DNA, ROS causes genomic damage and instability while in proteins, ROS inflicts harm through oxidative modifications. The combined adverse effects are termed oxidative stress. Oxidative stress has been implicated in the various hallmark capabilities of cancer (1-3). Research expanding several decades has demonstrated that antioxidants play a protective role in multistage carcinogenesis (1, 2). Generally, a living organism is equipped with protective enzymatic and non-enzymatic antioxidant mechanisms against ROS-induced oxidative damage. Nevertheless, these protective systems are insufficient to prevent the damage entirely (2). Recently, considerable attention has therefore been focused to identify synthetic antioxidants using natural phytochemicals as a substrate.

3-azabicylonanone is an important class of pharmacophore which has attracted the focus of attention of various medicinal chemists owing to their extensive scale of biological actions (4, 5). Previous research indicate that compounds with the thiadiazole ring possess a broad spectrum of biological activity (6-17). We had previously synthesized a set of 2r,4c-diaryl-3-azabicyclo [3.3.1] nonan-9-one-4-methyl-1,2,3-thiadazole-5-carbonyl-hydrazones by combining thiadiazole moieties with 3-azabicylonone in order to obtain higher bioactivities than 3-azabicylonone/ thiadiazole ring employed alone (18). Additionally, we documented the antimicrobial potency of the synthesized hydrazones derivative 9-15 against various bacteria and fungi (18). The current study highlights the *in vitro* antioxidant and antitumor effects against various free radicals and human cancer cell lines, respectively. Furthermore, a high level of p38α MAP kinase is correlated with highly invasive and proliferative phenotype of cancer cells (19-21). Therefore, we used bioinformatics tools to identify docking sites and confirm the interaction of synthetic compounds with p38*α *MAP kinase.

## Material and methods

**Chemistry**

**Fig. 1:**
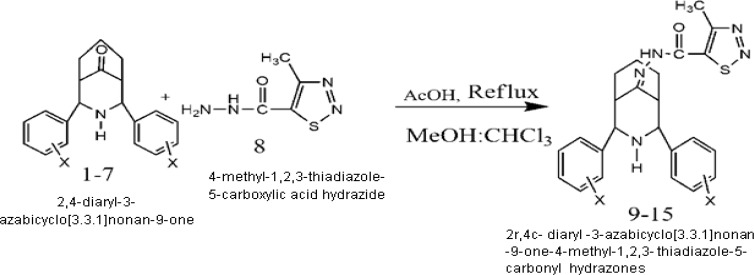
Synthesis of 2r,4c- diaryl -3-azabicyclo[3.3.1]nonan -9-one-4-methyl-1,2,3- thiadiazole-5-carbonyl hydrazones (9-15)

**Fig. 2:**
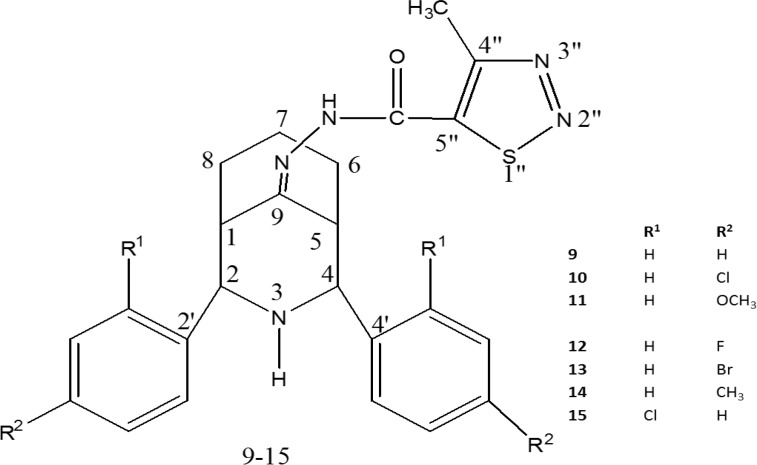
Numbering of the azabicycle [3.3.1] nonane

Synthesis of diversely substituted diaryl 3-azabicyclononan-ones 1-7 (22) and their methylthiadiazole hydrazones 9-15 were carried out according to the steps shown in figure 1. The compounds 9-15 were achieved by the reaction of the compounds 1-7 with 4-methyl-1,2,3-thiadiazole-5-carboxylic acid hydrazide 8, respectively (18). Definite structural elucidation has been carried out by exploring IR, H^1^, C^13 ^NMR and elemental analysis. 2D NMR spectra (^1^H-^1^H COSY, HSQC, HMBC and NOESY) recorded for a representative compound 12 confirmed the proposed structures for 9-15 (18). Figure 2 shows the numbering patterns of the compound. The substantial evidence for the proposed structure and twin-chair (CC) conformation of 2r,4c-diaryl-3-azabicyclo [3.3.1] nonan-9-one-4-methyl-1,2,3-thiadazole-5-carbonyl hydrazones 9-15 have been reported (18). The synthesized hydrazones 9-15 were screened for their antioxidant and anticancer effects.

***In vitro*** **Free Radical Scavenging Assays**

The free radical scavenging capacity was evaluated by the 2,2-diphenyl-1-picrylhydrazyl (DPPH) assay described by Blois (23). The total antioxidant potential was measured by the ABTS assay that measures the relative ability of the synthesized compounds to scavenge the ABTS^•+^ cation radical generated in the aqueous phase (24). Hydroxyl radical scavenging activity was determined by the method of Halliwell et al. (25) on the basis of the ability to compete with deoxyribose for hydroxyl radicals. The nitric oxide radical inhibition activity was evaluated according to the method of Nishimiki et al*. *(26). Superoxide anions derived from dissolved oxygen by a PMS/NADH coupling reaction reduced nitro blue tetrazolium (NBT) was measured by the method of Garrat using Griess reagent (27).

**Cell culture and maintenance**

HeLa cells derived from cervical cancer cells, adenocarcinomic human alveolar basal epithelial cells (A549) and *normal lung* epithelial cells (NL20) were obtained from the National Centre for Cell Sciences (NCCS), Pune, India. The cells were cultured in minimum essential medium, Dulbecco’s modified Eagle’s medium (DMEM), and Ham's F12 medium supplemented with 10% fetal bovine serum (FBS) (Sigma Chemical Co., St.Louis, USA), penicillin (100 U/ mL) and streptomycin (100 µg/ mL) as antibiotics (Himedia, Mumbai, India) in a humidified atmosphere of 5% CO_2_ at 37 ^o^C.

**Cell viability assay by MTT** **colorimetric** **assay**

Cell survival was assessed by 3-(4, 5-dimethy-lthiazol- 2- yl) -2, 5- diphenyltetrazolium bromide (MTT) assay. HeLa, A549 and NL20 cells, grown to approximately 80% confluence, were trypsinized, counted, seeded in 96-well plates with an average population of 1000 cells/ well, incubated overnight and then treated for 24 h with compounds 10, 12 and 13 at 0, 3, 6, 9, 12, 15, and 18 µM concentrations. MTT was added to each well and the plates were incubated at 37 C for 4 h followed by addition of 100 μL lysate (10% SDS in 0.01 mol/L HCl). The absorbance was measured at 490 nm using an ELISA microplate reader. All experiments were done in triplicates. Untreated cells were used as controls. 

**Cell proliferation assay by crystal blue staining method**

A549 and NL20 cells, grown to approximately 80% confluence were trypsinized, counted, seeded in 12-well plates with an average population of 40000 cells/ well, incubated overnight and then treated for 24 h with compounds 10, 12 and 13 at 8 μM concentrations. The cells were fixed and stained with crystal violet blue. The total cell number was recounted on day 1 and plotted for relative cell growth. All experiments were done in triplicate. Untreated cells were used as controls. 

**Cell migration assay**

HeLa cells were grown in 8 μM synthesized compounds 10, 12 and 13 for 24 h and plated into chamber with transwells the following day in medium containing 8 μM 10, 12 and 13 in triplicate as described by the manufacturer. The chamber transwell was taken out of the chamber 2 h after seeding, fixed and stained with DAPI. The number of cells per microscope field was generated by averaging 10 fields randomly selected. All counted cell numbers were used to plot the relative invasive potential.

**Molecular docking studies**

A docking study was carried out using Glide module of the Maestro software (Glide, version 6.0, Schrodinger, LLC, New York, NY, 2013) to investigate the detailed intermolecular interactions between the synthesized compounds and p38*α *MAP kinase. The 3D structure information on the target protein was taken from the PDB entry 3D83 (RCSB Protein Data Bank, http://www.rcsb.org/). The co-crystallized ligand was docked into the active site of p38*α *MAP kinase to validate the docking protocol. The protocol was set in which the best docking pose of the co-crystallized ligand showed all interactions that were reported in respective PDB. The same protocol for docking studies of synthesized compounds was followed. The errors like steric clashes, missing loops, missing atom names, etc... present in the 3D-structure of protein were corrected by the Protein Preparation module in Maestro (Maestro version 9.6, Schrodinger, LLC, New York, NY, 2013). The ligands were prepared (i.e., generation of possible ionization states, tautomers, stereoisomers etc...) using the Ligand Preparation Module in Maestro. An active site of 12 Å was created around the co-crystallized ligand. Extra precision (XP) mode and other default parameters of the Glide software were used for the docking studies.

**Statistical analyzes**

The data are expressed as mean± standard deviation (SD). The IC_50_ for *in vitro* antioxidant potential and MTT assay was calculated using linear regression analysis. Data for the anti-proliferative and anti-invasive effects of synthetic compounds were statistically analyzed using Tukey posthoc test. A probability value of less than 0.05 was considered significant. SD was made from three separate experiments.

## Results

**Free radical Scavenging activity**

Synthetic compounds 9-15 revealed a concentration- dependent antiradical activity resulting from reduction of DPPH^•^, ABTS^•+^, O^•-^, OH^•^ and nitric oxide radicals to their non- radical forms. IC_50_ values for the free radical scavenging effects of ascorbic acid and various synthetic compounds 9-15 are shown in Table 1. Compound 11 with electron-donor methoxy groups at the para position of the phenyl ring inhibited various free radicals by 50% at a concentration ranging from 6.93- 10.02 µg/ mL and showed highest antioxidant capacity compared to other compounds and standard antioxidant ascorbic acid, a known antioxidant used as a positive control. Treatment with synthetic compound 9 devoid of any substituents at the para position of the phenyl groups at the C-2 and C-6 positions of the azabicyclononan-9-one inhibited 50% of the various free radicals at the concentration ranging from 9.22- 14.01 µg/ mL. Compound 14 with electron-donor methyl groups at the para position of phenyl ring (7.45- 13.80 µg/ mL) also demonstrated higher antioxidant activity. Compounds possessing electron-withdrawing chloro (10), bromo (12/15) and fluoro (13), substitutions at the para/ ortho position of the phenyl ring (9.56- 15.33 µg/ mL) showed admirable *in vitro* free radical scavenging effects against various free radicals.

**Table 1:** IC_50_ valve for free radical scavenging activity (µg/mL).

**S.No**	**DPPH**	**ABTS**		**superoxide**	**Hydroxyl**		**Nitric oxide**
Ascorbic acid	7.50	9.52	10.19		12.03	13.32	
9	9.12	9.98	11.46		12.68	14.01	
10	9.56	10.16	11.79		12.78	14.19	
11	6.93	8.31	9.45		9.07	10.02	
12	10.12	10.96	11.83		12.94	14.82	
13	10.52	11.02	12.14		13.09	15.23	
14	7.45	9.96	11.06		12.43	13.80	
15	11.12	11.89	12.07		14.11	15.33	

IC_50_ values were determined by plotting dose-response curves of radical scavenging activities vs the concentration of synthetic compounds using GraphPad Prism version 4.00 for Windows (GraphPad Software Inc., San Diego, CA).

**Table 2:** IC_50_ valve for MTT assay (µmol)

**S.No**	**HeLa**	**A549**	**NL20**
9	9.91	11.28	12.35
10	4.41	6.61	12.36
11	10.27	12.56	13.45
12	4.43	6.60	8.63
13	4.48	4.61	8.36
14	10.01	12.43	13.02
15	10.03	12.56	13.25

IC_50_ values were determined by plotting dose-response curves of cytotoxic effects vs the concentration of synthetic compounds using GraphPad Prism version 4.00 for Windows (GraphPad Software Inc., San Diego, CA)

**Table 3:** The docking score and interactions (hydrogen bond and π-π interactions) for the synthesized compounds

**Comp-ound**	**Docking Score**	**Residue involv-ed in hydrogen bonds**	**Residues involv-ed in π-π intera-ctions **
**9**	-6.76	Asp-168	Lys 53, Arg 67
**10**	-6.70	Asp-168	Arg 67
**11**	-4.30	Asp-168	His 148, Arg 67
**12**	-5.29	Asp-168	-
**13**	-4.52	Asp-168	His 174, Arg 67
**14**	-4.69	Met-109	Phe 169
**15**	-5.19	Asp-168	Arg 67

**Anticancer effects**

All the synthesized compounds significantly inhibited the proliferation of cancer cells in a dose-dependent manner (0, 3, 6, 9, 12, 15 and 18 µM) after 24 h of incubation. IC_50_ values for the cytotoxic effects of various synthetic compounds 9-15 are shown in table 2. The highest activity was shown by the synthetic compound with electron withdrawing fluoro, chloro, and bromo functional groups (IC_50_= 4.43- 6.61 µmol) and the lowest by compounds with the electron donor functional groups (-CH3, -OCH3) present on the aryl rings attached to azabicyclononan-9-one moiety (IC_50_= 10.01- 12.56 µmol). The inhibitory effects of synthetic compounds were in the order: 10 > 12 > 13> 9> 14> 15> 11. 

**Fig. 3:**
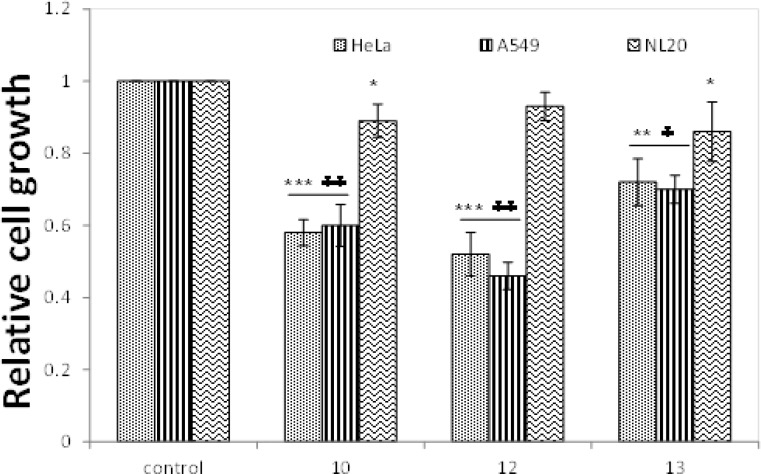
Antiproliferative effects of synthetic compounds 10, 12 and 13 at 8µM in HeLa A549 and NL20 cells by crystal blue staining method. * Significantly different from untreated control cells (P<0.05). ** Significantly different from untreated control cells (P<0.01). *** Significantly different from untreated control cells (P<0.001).   Significantly different from untreated NL20 cells (P<0.05).    Significantly different from untreated control cells (P<0.001).

Of the several synthetic compounds, compo-unds containing electron withdrawing functional groups (-F, -Cl and -Br) that were demonstrated to exert potent cytotoxic effects were used for testing their antiproliferative effects by crystal violet blue staining assay in comparison (Figure 3). Compo-unds 10 and 12 showed significantly (P< 0.001) greater inhibitory effect on HeLa and A549 cells compared to untreated control cells, whereas compound 13 displayed significantly (P< 0.01) higher cytotoxicity against tested cancer cells compared to untreated cells. The cytotoxicity of 10, 12 and 13 was also tested in normal lung epithelial cells (NL-20) near to the corresponding IC_50 _values. Accordingly, 10 and 13 displayed significant (P< 0.05) cytotoxic effect on cell viability in NL20 cells as compared to cancer cell lines (Figure 3) and untreated cells. However, no significant difference was observed in NL20 cells treated with compound 12.

**Cell migration assay**

**Fig. 4:**
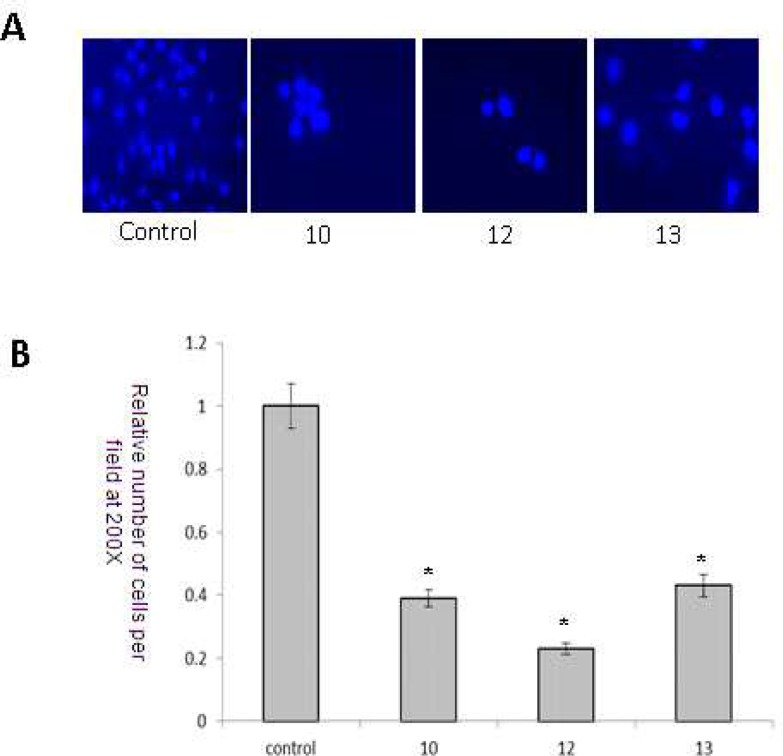
Anti-invasive potential of synthetic compounds 10, 12 and 13 (8 μM) by cell migration assay. * Significantly different from untreated control cells (P<0.05

The anti-invasive potential of synthetic compounds was examined by cell migration assay. Control cells have a stronger invasive potential as revealed by the increased number of cells (Figure 4 A and B). However, HeLa cells treated with compounds 10, 12 and 13 (8 μM) significantly (P< 0.001) mitigate the invasive potential of HeLa cells (Figure 4 A and B).

**Molecular docking study**

We have used bioinformatics tool to identify the interactions of newly synthesized compounds with docking sites on p38*α *MAP kinase. Molecular docking score may be used to predict the strength of association or binding affinity between synthetic compounds and p38α MAP kinase. Based on the molecular docking score, we revealed that synthesized compounds 9-13 and 15 interact with the catalytic domains of p38*α *MAP kinase to form hydrogen bonds with Asp 168 binding affinities ranging from −6.76 to −4.3 (Table 3).

**Fig. 5:**
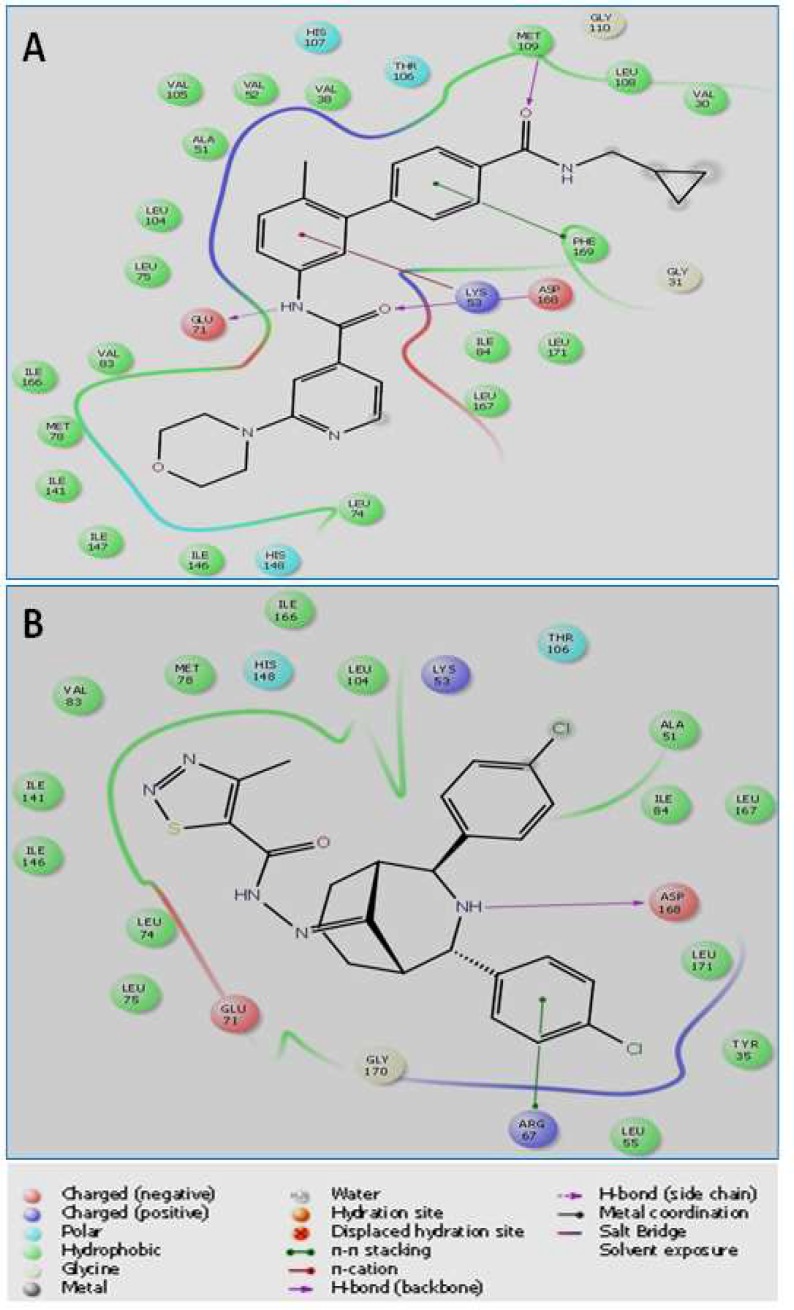
The interaction diagram showing hydrogen bonds and *pi*-*pi* interaction of (**A**) co-crystal ligand and (**B**) synthesized compound 10 with the active site of p38*α* MAP kinase

Figure 5 represents the interaction diagram showing hydrogen bonds and pi-pi interaction of (A) co-crystal ligand and (B-H) synthesized compounds (9-15) with the active site of the p38α MAP kinase. Compounds 9-13 and 15 interact with the p38α MAP kinase to form hydrogen bonds (green lines) with Asp-168 of p38MAPK. However, compound 14 interacts with the p38*α *MAP kinase to form a hydrogen bond with Met 109. Most of the synthesized compounds (9-11, 13, 15) show π-π interactions with Arg 67, and other residues like Lys 53 (9), His 148 (11), His 174 (13), Phe 169 (14). The binding affinity of synthetic compounds towards the active site of the p38*α *MAP kinase was in the order: 9 >10 > 12 > 15> 14> 13> 11.

## Discussion

Seven different hydrazone derivatives were synthesized and tested for their antioxidant potency by well-known *in vitro* antioxidant assays. DPPH^•^ and ABTS^•+^ radicals are known to accept an electron or hydrogen from the synthetic compounds to become stable non-radical forms. Under certain circumstances, O_2_ is reduced to H_2_O via O^•-^ and H_2_O_2_ and favors the formation of other reactive oxygen (OH^•^) and nitrogen (ONOO^-^) species (4, 5). Furthermore, H_2_O_2_ can be converted into hydroxyl radicals by the Fe^3+^-EDTA complex via the Fenton reaction (28, 29). These excessive production of toxic reactive oxygen (OH) and nitrogen (ONOO^-^) radical species are recognized to cause deleterious changes in DNA, lipid and protein oxidation. Thus, free radicals may serve as a source of mutations that initiate carcinogenesis (2).

Several studies have demonstrated that organic molecules incorporating an electron donor groups (amine, hydroxyl, methoxy and alkyl) at para position phenyl ring can act as free radical trapping agents and are capable of opposing oxidative challenges (30, 31). Consistent with these findings, phenyl rings with electron-donor methoxy and methyl groups at the para position of compounds 11 and 14 showed excellent free radical scavenging effects compared to standard antioxidant ascorbic acid, a known antioxidant used as a positive control. Compound 9, devoid of any substituents at the para position of the phenyl groups at the C-2 and C-6 positions of the azabicyclononan-9-one moiety, showed moderate *in vitro* free radical scavenging effects against various free radicals. Compounds possessing electron-withdrawing chloro, bromo and fluoro, substitutions at the para position of the piperidine moiety showed admirable *in vitro* free radical scavenging effects against various free radicals. This admirable free radical scavenging effects of compounds with nitro, bromo, choloro and fluoro substitutions may be due to the electron-withdrawing inductive effect of halogens. The results obtained in the present study are in line with other findings (32, 33). Compound 15 with an ortho chloro substituent in the phenyl moiety displays remarkable *in vitro* antioxidant activity. Taken together, the current research suggests that phenyl rings with electron-donor methoxy and methyl groups at the para position of compounds 11 and 14 with strong free-scavenging effects may conceivably contribute to its protective effects against free radical-induced oxidative stress and carcinogenesis. Therefore, further studies are warranted to establish its antioxidant and anti-carcinogenic effects using different experimental animal models.

We investigated the cytotoxic effects of newly-synthesized hydrazone derivatives on human lung cancer cell HeLa, A549, and NL20 cell growth using the MTT assay in order to validate their anticancer effects. Generally, compounds contai-ning electron withdrawing functional groups (-F, -Cl) exhibited more potent cytotoxic effects against the tested cancer cells compared to the electron donor functional groups (-CH3, -OCH3) present on the aryl rings attached to azabicyclononan-9-one moiety. Our results are in line with other research findings (34, 35). Contrary to reports of a positive correlation between the cytotoxicity and the antioxidant capacity of natural and synthetic compounds (35), we found low *in vitro *antioxidant activity in synthetic compounds 10, 12 and 13 despite high cytotoxicity. Strong cytotoxicity with poor antioxidant properties of synthetic compound 10, 12, and 13 may be ascribable to the pro-oxidant effects by the electron-withdrawing halogens. Although potent antioxi-dants often possess strong pro-oxidant activity, we found low cytotoxicity in synthetic compounds with electron- donating functional groups (-CH_3_, -OCH_3_) present on the aryl rings attached to azabic-yclononan-9-one moiety in line with the obser-vations of Lee et al. (36). However, multiple mechanisms regulate antioxidant and cytotoxic effects of the hybrid molecules, although they may contribute to the antioxidant activity and cytotoxicity to different degrees. Among the tested human cells, HeLa cells are more sensitive to all the synthetic compounds than A549 and NL20 cells.

Most of the compound induced significant cytotoxic effects in HeLa and A549 cells, although to different extents, (Table 2) the synthetic compounds containing electron withdrawing functional groups (-F, -Cl and -Br) exhibited more potent cytotoxic effects against cancer cells. Based on the IC_50_ value, these compounds, however displayed less cytotoxicity to NL20 normal lung epithelial cells. Particularly, the results of the compounds 10 seem to suggest a strikingly different effect on cancer and normal lung cells. Other compounds, which were toxic to lung cancer cells, were also toxic to lung normal cells to the more or less same extent.

Cell migration assay demonstrate that the synthetic compounds containing electron withdraw-ing fluoro, chloro and bromo functional groups can inhibit the growth and invasive potential of cancer cells and act as potent anticancer agent.

The p38*α *MAP kinases, serine/ threonine kinase p38 kinases, play a vital role in the regulation of cell growth, differentiation, apoptosis and responses to inflammation or stress (37, 38). In response to a variety of stress stimuli, p38α MAP kinase can be activated via dual phosphorylation of the TGY motif in the active site of the enzyme followed by phosphorylation of downstream substrates, thereby regulating various signaling pathways that are apparent in cancer (37, 38). Several studies have provided evidence that high levels of p38α MAP kinase was observed in highly invasive and proliferative phenotype of cancer cells (19-21). Pereira et al. documented that p38 MAPK inhibition results in ROS up-regulation, which in turn activates the JNK pathway via inactivation of phosphatases, sensitizing human tumor cells to cisplatin-induced apoptosis (39). Therefore, p38*α *MAP kinase inhibition would potentially inhibit invasive and proliferative effects of cancer cells. Molecular docking offers further insight in understanding the structure– activity relationship and binding modes of the query compounds.

Molecular docking study demonstrates that the synthesized compounds (9 and 10) have higher binding affinity for the active site of p38*α *MAP kinase, as compared to other compounds. Both the compounds have shown key interactions (H-bond and π-π) with the active site residues i.e. Asp 168, Lys 53, and Arg 67. Other compounds also have shown similar interactions with less docking score. In addition to H-bond and π-π interactions with active site residues, the docking score is also influenced by other interactions, including force field (electrostatic, van der Waals) contributions, water desolvation energy, and rewarding or penalizing interactions. This may be the reason for higher more docking score and binding affinity of synthetic compounds 9 and 10 to p38α MAP kinase compared to other compounds. Thus, our finding suggests that synthetic compounds may exert anti-proliferative and anti-invasive potential against various cancer cells through p38*α *MAP kinase inhibition. If these observations can be further confirmed through *in vivo* and *in vitro* experiments, p38*α *MAP kinase inhibition by the synthesized active hydrazone derivatives may be utilized to modulate key hallmark capabilities of cancer cells such as cell proliferation and apoptosis. Therefore, further studies are required to investigate the antitumor and p38*α *MAP kinase inhibitory effects of synthesized active hydrazone derivatives (9 and 10) in various human cancer cells and animal tumor models.
